# Empirically derived subgroups in rheumatoid arthritis: association with single-nucleotide polymorphisms on chromosome 6

**DOI:** 10.1186/1753-6561-1-s1-s20

**Published:** 2007-12-18

**Authors:** Marsha A Wilcox, Andrew T McAfee

**Affiliations:** 1 i3 Drug Safety, 950 Winter Street, Suite 3800, Waltham, Massachusetts 02431, USA

## Abstract

Rheumatoid arthritis (RA) is a disorder with important public health implications. It is possible that there are clinically distinctive subtypes of the disorder with different genetic etiologies. We used the data provided to the participants in the Genetic Analysis Workshop 15 to evaluate and describe clinically based subgroups and their genetic associations with single-nucleotide polymorphisms (SNPs) on chromosome 6, which harbors the HLA region. Detailed two- and three-SNP haplotype analyses were conducted in the HLA region. We used demographic, clinical self-report, and biomarker data from the entire sample (*n *= 8477) to identify and characterize the subgroups. We did not use the RA diagnosis itself in the identification of the subgroups. Nuclear families (715 families, 1998 individuals) were used to examine the genetic association with the HLA region. We found five distinct subgroups in the data. The first comprised unaffected family members. Cluster 2 was a mix of affected and unaffected in which patients endorsed symptoms not corroborated by physicians. Clusters 3 through 5 represented a severity continuum in RA. Cluster 5 was characterized by early onset severe disease. Cluster 2 showed no association on chromosome 6. Clusters 3 through 5 showed association with 17 SNPs on chromosome 6. In the HLA region, Cluster 3 showed single-, two-, and three-SNP association with the centromeric side of the region in an area of linkage disequilibrium. Cluster 5 showed both single- and two-SNP association with the telomeric side of the region in a second area of linkage disequilibrium. It will be important to replicate the subgroup structure and the association findings in an independent sample.

## Background

Rheumatoid arthritis (RA) affects nearly 1% of the population in the U.S. (2.5 million people). Symptoms vary from person to person and can include swollen and tender joints, pain, stiffness, and loss of motion. The symptoms have a range of presentation from intermittent flares to constant disabling pain [1].

Until recently, rheumatoid factor (RF) was the standard biomarker of severity in RA. According to the American College of Rheumatology, anti-cyclic citrullinated peptide (anti-CCP) antibodies are more specific than RF, may predict future RA in undifferentiated arthritis, are a marker for erosive disease, and may be an indicator of future disease in currently healthy patients [2].

The focus of this work was to identify groups of RA patients with similar clinical and biomarker characteristics. These subgroups, or clusters, were then examined for genetic association with the 404 single-nucleotide polymorphisms (SNPs) on chromosome 6. Chromosome 6 harbors the locus for the *HLA *gene, which has an established association with this disorder [3] and was chosen for that reason. Detailed analyses were conducted in the *HLA *region.

## Methods

This study was conducted in the sample from the North American Rheumatoid Arthritis Consortium (NARAC) data provided to the participants in Genetic Analysis Workshop (GAW) 15. The phenotypic subgroups were identified in the entire data set comprising 8477 individuals. There were 715 nuclear families with 1998 individuals available for the family-based association analyses.

Subgroups were identified on the basis of clinical and biomarker information. The data reduction method was based upon categorized data. There were 30 original variables with 112 associated categories. The following continuous variables were categorized for the analyses: age at onset, anti-CCP, RF, number of tender joints, number of swollen joints, joint alignment and motion score (JAM), severity of left and right hand erosions, and body mass index (BMI). The remainder of the variables used for clustering were retained their original coding in dichotomous categories: smoking, left or right hand erosions, physician and patient ARA ratings (morning stiffness, three or more joints groups swollen, arthritis of the hand joints, symmetric swelling, subcutaneous nodules, RF positive, x-ray changes with joint erosions). It is important to note that affectation status (unknown, unaffected, affected) for rheumatoid arthritis was omitted from the classification algorithm. The groups were formed using only clinical and biomarker indicators.

### Statistical methods

The strategy for the development of qualitative and quantitative traits included nonparametric data reduction, iterative two-staged clustering on the observed dimensions, and the assignment of binary cluster membership in each cluster for each individual.

Principal-components analysis (PCA) is a method commonly used for data reduction. These data did not meet the assumptions for PCA. A similar method designed for use with categorical data was employed. Multiple-correspondence analysis (MCA) is a nonparametric data reduction method free of the assumptions underlying PCA. The only requirement for MCA is a non-negative rectangular data matrix. MCA uses a singular value decomposition (SVD) of the matrix. Eigenvalue (vector) decomposition is a special case of SVD. The objective of MCA is to identify a low-dimensional subspace that comes closest to all of the data points. It is analogous to graphing the results of a factor analysis in a multidimensional Euclidean space. However, the space identified in MCA is not Euclidian. The coordinates of each individual in the identified multidimensional space served as the basis for the identification of subgroups or clusters [4].

Each study participant with phenotype data was assigned a score on each of the 20 retained dimensions. Next, a multistaged clustering strategy was used to identify distinct subgroups [5]. It is not unusual for groups identified with clustering techniques to be subject to the idiosyncrasies of the estimation data set. In an attempt to mitigate that difficulty, we first conducted repeated *k*-means clustering with different random cluster seeds and used a larger *k *(number of clusters) than we expected in the data. Groups that consistently clustered together across all of the initial analyses were identified as intact clusters. An agglomerative hierarchical clustering algorithm was then implemented using the intact clusters and the remaining individuals in the sample. An examination of the change in Ward's aggregation criterion and the nature of the groups was used to choose the final cluster structure [5,6]. We would have rejected a solution that contained, for example, a very small cluster formed primarily on the basis of data coding errors. The "corem", "defac", "recip/semis", and "parti/decal" modules of SPAD software [6] were used for both the multiple correspondence analysis and the clustering algorithms. SAS software [7] was used for subgroup comparisons.

There were four binary outcomes. Indicators of cluster membership in Clusters 2, 3, 4, and 5 were created for these analyses. Individuals were coded as belonging to one and only one cluster. The family-based test of association (FBAT) [8] was used to assess the association of subgroup (cluster) membership and the 404 SNPs on chromosome 6 and the two- and three-SNP haplotypes in the HLA region. Each cluster was analyzed separately; effectively comparing each cluster to all others. The two- and three-SNP analyses were conducted using a sliding window beginning at the telomeric side of the region. Analyses were conducted using the empirical variance due to the prior reports of linkage and association on this chromosome [9]. Linkage disequilibrium in the HLA region was estimated using Haploview version 3.32 [10].

## Results

### Correspondence analysis and clustering

Coordinates on 20 axes (analogous to factor scores) were retained and used for clustering. Twenty axes were retained based upon an examination of a graph of the eigenvalues. This strategy accounted for 74% of the original variance in the data. The first axis was characterized by patient and physician reports of stiffness and swelling, arthritis in the hands, three or more joint groups swollen and, erosions. The second axis was characterized by physician ratings on the ARA scale and the presence of subcutaneous nodules. A combination of the self-report variables, particularly the physician reports, and clinical symptoms were more influential in the formation of the first two axes than were the biomarker data.

Five groups were identified as a result of data reduction and clustering. Cluster sizes were: Cluster 1, *n *= 6,583 (77.7%); Cluster 2, *n *= 392 (4.6%); Cluster 3, *n *= 558 (6.6%); Cluster 4, *n *= 513 (6.1%); Cluster 5, *n *= 431 (5.1%). Cluster 1 was composed entirely of unaffected individuals and will not be considered further in the description of the RA subtypes. The distribution of Clusters 2 through 5, omitting the large unaffected cluster, is: 20.7%, 29.5%, 27.1%, and 22.8%. Table 1 shows the distribution of clinical indicators and biomarkers for Clusters 2 through 5, with an indication of statistical differences between clusters.

**Table 1:** Cluster characteristics and differences between clusters

Trait	Cluster 2	Cluster 3	Cluster 4	Cluster 5
Rheumatoid arthritis (% affected)	44.4	99.1	99.8	100
Anti-CCP	*^a^	52.3	124.8^3^	127.8^3^
Rheumatoid factor	*	33	111^2,3^	138^2,3^
Joint alignment and motion score	*	5	21.5^3^	65^2,3,4^
				
Age at onset (median)	36	42^2,5^	40^2,5^	32
Female (%)	69.1	74.7	74.1	84.5
BMI (median)	24.0	26.6^5^	25.1	24.8
				
Subcutaneous nodules (%)	16.3	22.4	54.4	68.0
R/L hand erosions (%)	*	73.5	100	100
Severity R/L hand (5-point scales)	*	2	3^3^	5^2,3,4^
Tender joint count	*	4	6^3^	6^3^
Swollen joint count	*	5	8^3^	8^3^

Superscripts indicate clusters compared to which the column cluster is significantly higher. For example, the JAM score for Cluster 5 is significantly higher than Clusters 2, 3, and 4.

*^a^, Data are missing for the majority of this cluster.

Cluster 2 is an interesting mix of unaffected and affected individuals. Less than half are "affected" with RA. This group is distinguished by a pattern of patient endorsement of the ARA symptoms (arthritis in the hand joints, three or more swollen joint groups, symmetric swelling, morning stiffness, and RF positive) and a failure of physician corroboration of the same list of symptoms. Of clinical note, a smaller proportion of this group has subcutaneous nodules than the other groups.

Clusters 3 through 5 represent a severity continuum of rheumatoid arthritis. Almost all of the individuals in each of these clusters are affected with the traditional diagnosis of RA. Cluster 3 has significantly lower scores than Clusters 4 and 5 for anti-CCP, RF, and the JAM score. The BMI of Cluster 3 members is slightly higher than that of members of the early onset severe disease Cluster 5. Fewer members of this group report left or right hand erosions (74% compared with 100% in Clusters 4 and 5). They have fewer tender or swollen joints and have lower ratings on the severity rating scales. They are mildly affected.

Cluster 4 is a moderately affected group. The manifestation of disease in this group is generally more severe than in Cluster 3. Disease severity in Cluster 4 is closer to the severely affected Cluster 5 than to the mildly affected Cluster 3. The JAM score for this group is significantly higher than Cluster 3 and significantly lower than Cluster 5. They are also significantly higher than Cluster 3 on RF, severity ratings, and the number of swollen and tender joints.

Cluster 5 is characterized by earlier onset severe disease. This group has significantly earlier onset than any of the other clusters. The highest anti-CCP, RF, and JAM values are observed in this group. There are more women in this group (85% compared with 69%, 75%, and 74%). The median severity score is 5 on a 5-point scale. More than two-thirds of this group have subcutaneous nodules (68% compared with 22% and 54%).

There is a continuum from Cluster 3 to Cluster 5 for JAM score, x-ray changes with erosions, subcutaneous nodules, and to a lesser degree, proportion RF positive.

### Association

**Figure 1:**
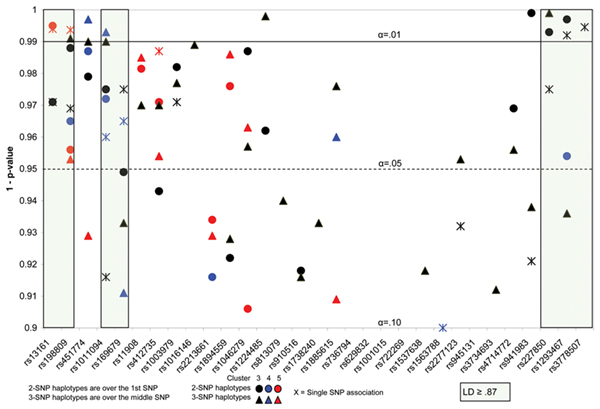
**Single-, two-, and three-SNP haplotype association with Clusters 3, 4, 5**. The y-axis shows 1-*p*-value with reference lines for α = 0.05 and α = 0.01. Circles represent the two-SNP analyses, triangles the three-SNP. Cluster 3 results are in black type, Cluster 4 in blue, and Cluster 5 in red. The shaded areas on either side of the region show strong linkage disequilibrium (greater than 0.86).

The single-SNP analysis focused on the SNPs on chromosome 6 because the HLA locus is on 6p and has been shown to be influential in the etiology of RA. We further examined the HLA region with haplotype analyses. FBAT results are presented in Table 2 for SNPs and clusters outside of the HLA region where the *p*-value was 0.01 or lower. Results of the single-, two-, and three-SNP analyses with *p*-values less than 0.10 in the HLA region are presented in Figure 1. This level was chosen to facilitate the examination of patterns of association across the region and among the clusters. Cluster 3 shows single-, two-, and three-SNP association (*p *< 0.01) in a strong LD region at the centromeric side of the region. Cluster 5 shows single- and two-SNP association (*p *< 0.01) in the telomeric side of the region.

**Table 2:** FBAT results for SNPs outside the HLA region

SNP	Gene	Location	Informative families	*Z*	*p*-Value^a^	Cluster
rs6597161	*FARS2*	6p25.1	157	2.58	0.0098	3
rs1953088	*PHACTR1*	6p24.1	102	3.91	0.0001	5
rs7775474		6p23	107	2.69	0.0071	5
rs1938115		6q12	136	2.85	0.0043	3
rs2796051	*KCNQ5*	6q14	16	2.95	0.0032	5
rs6907703		6q14	105	2.84	0.0046	4
rs1548297	*EPHA7*	6q16.1	127	2.73	0.0063	4
rs1158747	*LAMA4*	6q21	144	2.64	0.0084	3
rs2030926		6q22	156	3.26	0.0011	3
rs1012509	*MAN1A1*	6q22	95	2.79	0.0053	5
rs577876	*PARK2*	6q25.2	134	3.77	0.0002	3
rs880900	*RPS6KA2*	6q27	86	2.64	0.0084	5
rs6917113		6q27	72	3.00	0.0027	5

^a^Values less than 0.01 are shown.

## Discussion

We identified five empirically derived, phenotypically distinct subgroups. One group was unaffected; one was characterized by patient complaints without physician corroboration. Three comprise a severity continuum of rheumatoid arthritis. The distinguishing features of the most severely affected group are younger age of onset, more subcutaneous nodules, significantly higher JAM scores, slightly higher RF values, and more radiological changes with erosions.

The cluster with the earliest age of onset and most severe disease shows association (*p *< 0.01) with 8 SNPs on chromosome 6. The strongest association is with *PHACTR1*, phosphatase and actin regulator 1, a gene thought to interact with actin alpha and skeletal muscle 1 [11].

Clusters 3 and 5 are different from one another clinically and in association with the HLA region. The large number of tests conducted in this study (404 SNPs with 4 traits, or 1616 tests) makes it possible that these findings are due to chance alone. One would expect about 16 such results at α = 0.01; we report 13. However, the purpose of this research was hypothesis generation. It will be important to conduct hypothesis testing to assess the validity and utility of this characterization of RA.

## Competing interests

The author(s) declare that they have no competing interests.
